# Role of Endometrial Extracellular Vesicles in Mediating Cell-to-Cell Communication in the Uterus: A Review

**DOI:** 10.3390/cells12222584

**Published:** 2023-11-07

**Authors:** Jacob R. Beal, Qiuyan Ma, Indrani C. Bagchi, Milan K. Bagchi

**Affiliations:** 1 Department of Molecular and Integrative Physiology, University of Illinois at Urbana-Champaign, Urbana, IL 61801, USA; 2 Department of Comparative Biosciences, University of Illinois at Urbana-Champaign, Urbana, IL 61801, USA

**Keywords:** extracellular vesicles, pregnancy, endometrium, angiogenesis, maternal–fetal interface

## Abstract

There are several critical events that occur in the uterus during early pregnancy which are necessary for the establishment and maintenance of pregnancy. These events include blastocyst implantation, uterine decidualization, uterine neoangiogenesis, differentiation of trophoblast stem cells into different trophoblast cell lineages, and formation of a placenta. These processes involve several different cell types within the pregnant uterus. Communication between these cell types must be intricately coordinated for successful embryo implantation and the formation of a functional maternal–fetal interface in the placenta. Understanding how this intricate coordination transpires has been a focus of researchers in the field for many years. It has long been understood that maternal endometrial tissue plays a key role in intercellular signaling during early pregnancy, sending signals to nearby tissues in a paracrine manner. Recently, insights have been obtained into the mechanisms by which these signaling events occur. Notably, the endometrium has been shown to secrete extracellular vesicles (EVs) that contain crucial cargo (proteins, lipids, RNA, miRNA) that are taken up by recipient cells to initiate a response leading to the occurrence of critical events during implantation and placentation. In this review, we aim to summarize the role that endometrium-derived EVs play in mediating cell-to-cell communications within the pregnant uterus to orchestrate the events that must occur to establish and maintain pregnancy. We will also discuss how aberrant endometrial EV signaling may lead to pathophysiological conditions, such as endometriosis and infertility.

## 1. Introduction

During mammalian pregnancy, the uterus is responsible for supporting the growth and development of the fetus. For successful pregnancy establishment, several important processes must occur within the uterus to enable the fetus to receive essential nutrients and allow for the development of an interface for gas and waste exchange. The first major event that needs to occur is implantation of the embryo, which requires attachment of the embryo to the uterine endometrial luminal epithelium followed by invasion deeper in the endometrial stroma below [1,2]. At this point, crosstalk between the endometrial epithelium, underlying stroma, and the implanting embryo is critical to ensure successful implantation, as well as to begin to prepare the uterus to support proper embryonic development [3,4]. Embryonic invasion into the stroma subsequently triggers another crucial process, which is the ovarian steroid-mediated transformation of the endometrial stroma into a secretory tissue termed the decidua [5,6,7,8]. This tissue is then responsible for the production and secretion of various paracrine factors that regulate additional important processes, such as angiogenesis and placentation, that must occur within the uterus during pregnancy [9,10].

After implantation, as the embryo begins to develop, increased blood supply to the uterus is needed to support the growing fetus [11,12]. Adaptation of the uterine vasculature to meet this increased demand is coordinated by paracrine signals sent by the endometrial decidua to develop an intricate angiogenic network within the uterus during the early days of pregnancy [9,13]. This angiogenic network supports the fetus as the placenta begins to form and vascularize. 

Maternal–fetal crosstalk is also vital for the formation of a functional placenta. Spatio-temporal coordination of trophoblast differentiation and invasion and uterine remodeling must be precise for the placenta to form correctly. In humans, cytotrophoblasts (CTs) can differentiate into two main subtypes, multinucleated syncytiotrophoblasts (STs) and invasive extravillous trophoblasts (EVTs), and they must do so in the appropriate ratio to allow the EVTs to invade and anchor the placenta to the decidua [14,15]. The endometrial decidua produces secretory factors that both promote and inhibit trophoblast invasion, consistent with the need to allow the progress of trophoblast invasion while showing some restraint in this process [16,17,18,19].

The significance of the role that endometrial paracrine signaling plays is apparent in all these crucial events during early pregnancy. Any interruption to these signaling pathways has been shown to impair the development of the placenta, which results in various diseases, such as intrauterine growth restriction, preeclampsia, and recurrent miscarriage [20,21,22,23,24,25]. Unsurprisingly, it has been a major goal in reproductive sciences to better understand the mechanism by which the endometrium can send these signals to other cells within the uterine environment to direct them to enact such vital adaptations.

In recent years, there has been an increased focus on studying how extracellular vesicles (EVs) act as key mediators of cell–cell communication. EVs are membrane-enclosed vesicles that are secreted into the extracellular space by many different cell and tissue types, including several tissues that play crucial roles during early pregnancy [26,27]. EVs have been found to be secreted by the embryo [28], placenta [29], endometrial stromal [30] and epithelial cells [31], and oviductal epithelial cells [32]. These EVs are shed by one cell and can be taken up by another. They often contain DNA, RNA, proteins, and lipids as cargo that can act as signaling molecules that are transferred from the secreting to the recipient cell, inducing a functional change in the latter. This allows EVs to act as a vehicle for communication between two different tissues within the uterus during pregnancy, as well as intratissue communication.

EVs are sometimes classified into subgroups based on their size and differences in biogenesis pathway. EVs that are 200 nm–1 μm in size are often termed microvesicles (MVs), whereas vesicles that are 40–200 nm in size are considered small-EVs. This distinction is made because of the different biogenesis pathways that each subset uses. MVs are secreted directly by the budding of the plasma membrane. Small-EVs are formed within the endosomal compartment made via endocytosis of the plasma membrane to form the early endosome, followed by repeated inward budding of the early endosome to form a late endosome, or multivesicular body (MVB). The MVB is then trafficked to the plasma membrane, where it fuses and releases its contents, the small-EVs, into the extracellular space [33]. However, it is often difficult to isolate and individually study small-Evs and MVs, and the nomenclature used for reporting EV research has been inconsistent over the years. Therefore, in this review, while we will consider research that specifies MVs or small-EVs, we will refer to all vesicles as EVs, as is the consensus recommendation by the International Society for Extracellular Vesicles [34].

**Figure 1:**
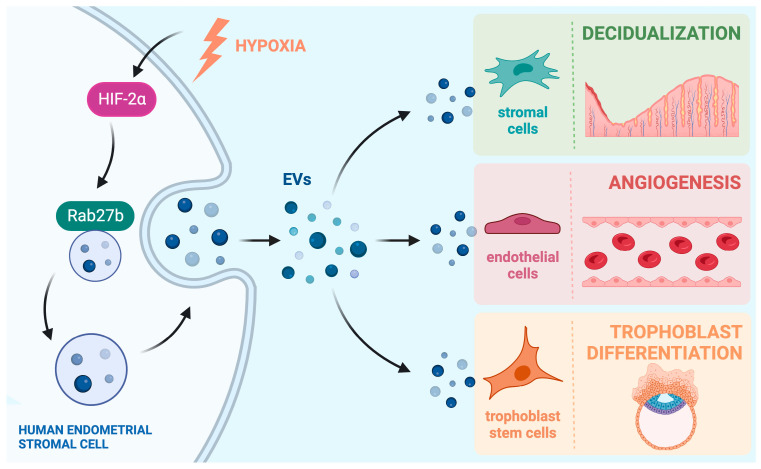
**Endometrial EVs mediate cell-to-cell communication within the uterus.** Production of EVs via differentiating human endometrial stromal cells is regulated by the hypoxia-inducible transcription factor HIF2α and its downstream target Rab27b, which controls vesicular trafficking. The EVs secreted by the stromal cells are taken up by stromal, endothelial, and trophoblast cells in the uterine milieu to induce changes in their functions. In this way, EVs play a vital role in supporting decidualization, blood vessel formation, and trophoblast development, which are integral parts of implantation and placentation during early pregnancy. Created in BioRender.com (10 July 2023).

In this review, we will summarize important recent findings on how EVs secreted from the endometrial cells mediate cell-to-cell communication within the pregnant uterus and influence a variety of functions at the maternal–fetal interface. We will also discuss findings that show how aberrant endometrial EV signaling may lead to various pathophysiological conditions that occur in the uterus. A summary of endometrial EV signaling pathways can be seen in Figure 1.

## 2. EV-Mediated Cell-to-Cell Communication within Endometrium Alters Critical Uterine Functions

In the pregnant uterus, ovarian steroid hormones estrogen and progesterone influence a remarkable transformation of endometrial stromal cells that allows them to support embryo growth and maintain early pregnancy. This transformation process is known as decidualization, and it is seen in many mammals, including humans. A hallmark of this process is the increased secretory nature of the differentiated endometrial stromal cells that create the transient tissue called the “decidua”. As such, it comes as no surprise that during the decidualization process, there is an increased number of EVs secreted by the endometrial stromal cells. This increase in EV production has been shown in both mouse [35] and human decidual cells [30]. Ma et al. showed that, in both species, EV secretion by decidual cells is regulated by a conserved pathway, which consists of the transcription factor hypoxia inducible factor 2 alpha (HIF2α), which consequently controls the expression of the vesicular trafficking protein RAB27B, which is one of the proteins responsible for guiding the MVB from the cytosol to the membrane for secretion [30,35]. In a separate study in mice, conditional ablation of HIF2α in the uterus led to a downregulation of RAB27B protein and consequently showed a perinuclear accumulation of vesicles that were unable to be transported to the membrane to be secreted [36]. 

This increase in endometrial EV production allows for crucial communication between the cells within the implantation chamber and during placenta development (Figure 1). Broadly, the mammalian endometrium is made up of two regions, the luminal epithelium, which makes up the uterine lining, and the stromal compartment, which contains stromal cells, glands, blood vessels, and immune cells. Both endometrial epithelial and stromal cells have been shown to secrete EVs, and it is vital that the two compartments communicate with each other in a paracrine manner, as well as with themselves in an autocrine manner through EV signaling [30,31].

The decidualization of the endometrial stroma is an important step in increased EV secretion during pregnancy. Mechanical stimulation by the blastocyst reaching the endometrium, as well as estrogen and progesterone signaling, initiate the decidualization process [37]. Continued decidualization of endometrial stromal cells is, of course, still regulated by the ovarian hormones; however, Ma et al. have shown that EVs produced by decidualizing endometrial stromal cells contain protein cargoes that may promote further decidualization. EVs were shown to contain proteins such as glucose transporter 1 (GLUT1) and pyruvate kinase (PKM), both of which are known to be metabolic regulators that can influence the decidualization process [30,35]. Other potentially relevant protein cargoes were found, and a partial list of the cargoes found in these studies and others referenced in this review can be found in Table 1. The authors also confirmed via confocal microscopy that endometrial stromal cells were able to uptake fluorescently labeled EVs collected from the conditioned media of decidualizing cells. They showed that upon uptake of additional endometrial EVs, there was an increase in markers of decidualization in the recipient cells [30,35]. These data indicated that via autocrine EV signaling or paracrine signaling to nearby cells, endometrial EVs are able to increase decidualization in neighboring endometrial stromal cells, forming a positive feedback loop and ensuring a tightly controlled spatial progression of decidualization that is critical for the establishment of an environment conducive to embryonic growth. 

During implantation, before embedding into the decidua, the blastocyst must first attach to the endometrial luminal epithelium. EVs that are taken up by the endometrial epithelium have been shown to be an important part of this process, allowing for the endometrium to become receptive to the blastocyst. The exact origin of these EVs is not always clear in some studies. In larger mammals, such as livestock, characterization of uterine flushing fluid-EVs (UF-EVs) is a common practice to study the effect of EVs during the peri-implantation period [46]. UF-EVs may contain EVs that originate from either the embryo or endometrium.

Bidirectional communication between the endometrium and the fetus is critical for successful implantation and is a major area of study. In this review, for the sake of brevity, we will discuss endometrium-to-fetus EV signaling, but not the reverse. However, we can assume that during the peri-implantation period, the majority of EVs come from the endometrium, due to the much larger quantity of endometrial cells that produce EVs compared to the blastocyst. Also, Hu et al. performed immunofluorescence analysis of a porcine uterus to show that EV-specific markers are primarily found in the epithelium throughout the implantation window but are only seen in the conceptus on later days, suggesting that the majority of UF-EVs originate from the endometrial epithelium and can be transferred to the embryo, presumably via direct uptake [47].

In sheep, Burns et al. showed that EVs isolated from the uterine luminal fluid (ULF) can be taken up by endometrial epithelial cells [46]. Similarly, Hua et al. found that, in pigs, UF-EVs can be taken up by epithelial cells in culture. They also went on to show that the addition of UF-EVs to primary endometrial epithelial cells vastly altered the transcriptome of the epithelial cells. RNA-sequencing was performed on the cells to determine any changes to their transcriptome. The mRNA expression levels of 690 genes were significantly up-regulated, and 1103 genes were significantly down-regulated. Several of the differentially expressed genes are related to embryo implantation, such as members of the matrix metallopeptidase (MMP,) interferon (IFN), insulin-like growth factor (IGF), and cell adhesion signaling pathways [42]. Previous work in the same lab had shown that UF-EVs contained miRNAs that were differentially expressed during the pre- and peri-implantation period in pigs [48]. These miRNAs included ssc-let-7a and let-7g, which had previously been shown to enhance endometrial receptivity by suppressing the Wnt pathway in mice and humans [49]. Together, these data showed how changing the miRNA profile of UF-EVs during the implantation period affects the transcriptome of the endometrial epithelium to allow for increased endometrial receptivity to allow blastocyst implantation.

## 3. EV-Mediated Communication Is Critical for Maternal–Fetal Interactions

Communication between the maternal endometrium and the fetus is vital for the duration of the pregnancy. It is necessary for proper blastocyst implantation and throughout the placentation process. Some of the earliest work characterizing endometrial EVs showed the potential for communication between the endometrium and embryo. 

In 2013, Ng et al. showed the presence of EVs in the uterine cavity for the first time [38]. The authors also isolated EVs produced by the endometrial epithelial cell line ECC1 and characterized the miRNA stored within these EVs. They identified several miRNAs, including hsa-miR-484, hsa-miR-92a, and hsa-let-7e. By analyzing the predicted target genes of all miRNAs found in their EVs, the authors showed that these miRNAs had predicted effects on genes known to be members of several pathways important to implantation, such as adherens junctions, ECM-receptor interaction, Jak-STAT, and VEGF-signaling pathways [38]. Vilella et al. later profiled maternal miRNA that are differentially expressed in the endometrial epithelium during the window of implantation [35]. Some of these miRNAs were found to be secreted into the endometrial fluid to be transferred to the blastocyst. Specifically, hsa-miR-30d was found within secreted EVs, and these EVs were shown to be internalized by the mouse trophectoderm. This potentially led to an increase in expression of genes involved in murine embryonic adhesion, such as ITGB3, ITGA7, and CDH5 [39].

A later paper by Greening et al. looked at the protein cargoes of the endometrial epithelial EVs [50]. Similar to the miRNA findings by Ng et al. [38], they identified several protein cargoes that play key roles in implantation-related pathways, such as adhesion, migration, and invasion. These proteins include ADAMTS15, HSPG2, and EGFR, amongst others. They went on to show that the endometrial epithelial EVs can be taken up by trophoblast cells and produce a functional change within the trophoblast, enhancing their adhesive capacity, which is critical during normal implantation. 

Importantly, the authors compared EVs collected from cells treated with only estrogen (emulating the nonreceptive phase) and cells treated with estrogen and progesterone (emulating the receptive phase) and discovered that some protein cargoes were found to be enriched only after addition of progesterone [50]. The presence of progesterone mimics the hormonal levels during the window of implantation and suggests an important role for hormonal regulation of EV signaling during pregnancy. Other studies have shown a similar importance of progesterone regulation of EVs. Burns et al. ovariectomized ewes and used hormone replacement to determine the effect of progesterone. They found that not only did progesterone regulate the presence of several key miRNAs in EVs, but progesterone also altered the quantity of EVs produced by the endometrium. In the absence of progesterone signaling, significantly fewer EVs were released into the uterine cavity [51]. Ma et al. [30] also found that progesterone increased endometrial EV secretion, this time in primary human endometrial stromal cell culture. EV concentration in the conditioned media was doubled in response to progesterone compared to the control and was further increased upon the addition of the entire decidualization cocktail, consisting of estrogen, progesterone, and 8-Bromo-cAMP.

In the Ma et al. [30] study, the authors also showed an important interaction between endometrial EVs and the fetus that occurs after implantation. During human placentation, cytotrophoblast cells need to differentiate into the invasive EVT lineage to embed deeper into the decidua and remodel spiral arteries to provide nutrients to the placenta. The authors showed that the differentiation process of cytotrophoblast cells to invasive EVTs was enhanced by the addition of endometrial stromal-derived EVs to the culture. This finding was consistent with their data identifying several protein endometrial EV cargoes that are potential regulators of trophoblast differentiation, including members of the IGF-signaling (IGFBP1, IGFBP3, IGFBP5, IGFBP7, IGF2) and TGFB-signaling (TGFBI, TGFB1) families [30]. 

Liu et al. also investigated the effect of endometrial stromal EVs on the trophoblast invasion process [52]. They showed that EVs isolated from decidualized endometrial stromal cells can be taken up by trophoblast cells and that these trophoblasts resultantly showed an increased level of invasiveness. Interestingly, they showed that these phenomena occurred via the upregulation of N-Cadherin expression in the trophoblast cells, and the increased invasiveness phenotype could be blocked by the silencing of N-cadherin. They also showed that N-cadherin expression increased due to elevated levels of SMAD2/3 in the trophoblasts in response to the addition of endometrial EVs [52].

The interaction between endometrial EVs and invading trophoblasts has also been described using a porcine model, though this time the source of endometrial EVs was epithelial cells instead of the stroma. Hu et al. showed that ULF-EVs which originated from endometrial epithelial cells can be taken up by porcine trophoblast cells. Interestingly, EVs isolated during the beginnings of implantation (Day 9 of pregnancy) when added to trophoblast culture promoted the migration of the trophoblasts in a transwell assay, although this difference was statistically insignificant. However, the addition of EVs isolated from later days of pregnancy (Day 12 and Day 15) significantly inhibited the migration of the trophoblasts [47]. Pigs have a non-invasive epitheliochorial placentation [53], so the ability of EVs in this case to promote trophoblast migration early during implantation allows for blastocyst attachment. Later, they inhibit migration to prevent further invasion into the endometrium, which is an essential characteristic of porcine implantation.

In 2014, Burns et al. showed that, in sheep, ULF-EVs contain endogenous beta retroviruses (enJSRVs) as cargo which are understood to be transferred from the endometrial epithelia to the conceptus trophectoderm to aid in its development [54]. They also identified several miRNAs with potential effects on the implantation process when delivered to the fetus. These miRNAs include a few that appear to be conserved across species, as they were also found in porcine (let-7a [48]) and human (miR-30d [38]) endometria. The same group later confirmed that ULF-EVs can be taken up by the conceptus trophectoderm [46], further underlining the importance of EVs in the communication between the endometrium and conceptus during pregnancy. Ruiz-Gonzalez et al. also showed a similar relationship between enJSRVs, EVs, and endometrium–trophectoderm communication in sheep. They showed that UF-EVs contained enJSRVs and that addition of the EVs to conceptus trophectoderm cells induced the cells to proliferate and secrete interferon-tau (IFNT) in a dose-dependent manner [40]. IFNT is an important signal necessary for maternal recognition of pregnancy in ruminants and exclusively secreted by cells of the trophectoderm [55].

## 4. EVs Influence Maternal Angiogenesis and Blood Vessel Formation

Uterine angiogenesis plays a vital role during early pregnancy. During angiogenesis, new blood vessels are formed from pre-existing ones, generally by sprouting or splitting off the parent vessel. Endothelial cells make up the walls of these vessels and are receptive to various chemical signals which instruct them to create these new blood vessels [56]. EVs are well documented to contain many of these chemical signals that induce angiogenesis, such as growth factors and chemokines [57,58,59]. 

Increased blood flow to the uterus to provide nutrients to the growing embryo is vital during pregnancy, so uterine angiogenesis must be tightly regulated within the implantation chamber and during placentation. The endometrium plays a large role in promoting uterine angiogenesis via EV signaling to the endothelial cells of nearby blood vessels. Ma et al. showed that fluorescently labeled EVs derived from decidualized primary human endometrial stromal cells can be taken up by primary endothelial cells in culture. Addition of the endometrial EVs increased proliferation of the endothelial cells and increased the expression of the angiogenic marker angiopoietin-2. They also showed a functional effect with the EV-enhanced endothelial cells, showing an increased ability to form capillary-like structures in an in vitro tube formation assay. Again, several essential EV protein cargoes were identified that have known angiogenic effects; these include RAC1, ANGPT1, ANGPTL2, GJA1, and MMP2, to name a few [30].

Harp et al. showed that EVs derived from endometrial stromal cells contain miR-21 and miR-126, which are two miRNAs that have been previously identified as pro-angiogenic. They established that the addition of endometrial stromal EVs in primary endothelial cells induced tube formation and that tube formation increased as levels of miR-21 within EVs increased in response to different conditions [41]. 

Endometrial mesenchymal stem cells (endMSCs) lie within the stromal compartment and are the progenitor cells of endometrial stomal and epithelial cells. As well as existing as a source for renewal for endometrial stromal and epithelial cells, endMSCs play an immunomodulatory and pro-angiogenic role within the uterus [60]. Marinaro et al. found that EVs derived from endMSCs contained the protein ERAP-1 [45]. This protein could play dual roles in modulating the immune response and promoting angiogenesis. ERAP-1 is known to increase the shedding of cytokine receptors, modulating the overall immune response [61]. ERAP-1 also promotes proliferation and migration of endothelial cells upon stimulation with VEGF [62].

Nooshabadi et al. also showed the angiogenic potential of endMSC-derived EVs. They treated human endothelial cells with endMSCs-EVs and proved that the endothelial cells could uptake the EVs. Furthermore, the endMSC-EVs increased the proliferative, migratory, and angiogenic capabilities of the recipient cells, as shown by in vitro assays [63].

## 5. Aberrant EV Signaling Is Associated with Endometrial Dysfunction

**Figure 2:**
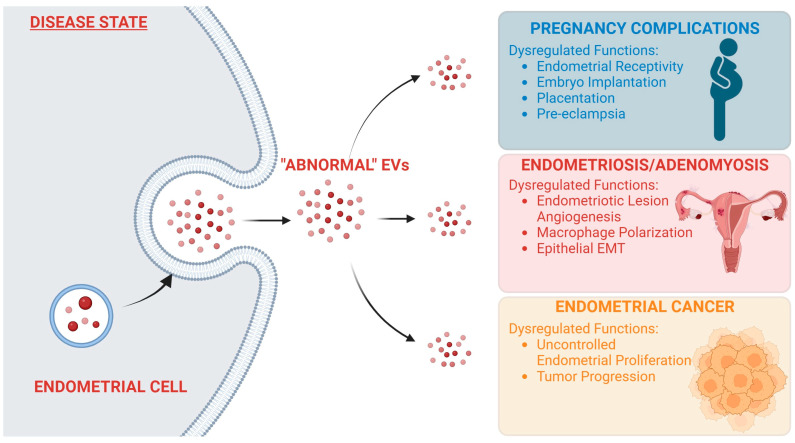
**“Abnormal” endometrial EV signaling leads to development of uterine disorders.** Endometrial EV signaling is necessary for healthy uterine functions. Cargoes carried by EVs regulate important functions in recipient cells. In a disease state, EV cargoes can be altered, leading to “abnormal” EVs being secreted by endometrial cells. When “abnormal” EVs are taken up by recipient cells, this can cause dysregulation of several major functions, leading to the development of various gynecological conditions. Created in BioRender.com (24 October 2023).

EVs are a powerful mechanism for signaling within the body, and as such, tight regulation of their synthesis and secretion is crucial for normal body function. Any changes from normal EV signaling can be the cause of deleterious effects throughout the body. EV signaling and its role in cancer progression and metastasis have been well documented [64,65,66]. Aberrant EV signaling has also been linked to neurodegenerative disorders [67], diabetes [68], and rheumatoid arthritis [69], among other pathophysiological conditions. Unsurprisingly, dysregulation of endometrial EV signaling also can lead to several pathophysiological conditions in the reproductive tract (Figure 2).

### 5.1. Endometriosis

Endometriosis is a disease where endometrial tissue grows outside of the uterus. It is benign, but symptoms can include pelvic pain, menstrual irregularities, and infertility in some affected women [70]. Its etiology is still not completely understood, but one of the most commonly accepted theories is Sampson’s theory of retrograde menstruation, which was posited in 1927. The theory suggests that endometrial tissue travels retrogradely through the Fallopian tube and is deposited outside the uterus at sites where it can establish a link to blood supply, allowing for proliferation of the ectopic tissue [71]. Angiogenesis obviously would play a crucial role in this theory, coordinating the development of new blood vessels to establish a blood supply. We have already discussed the relationship between endometrial EVs and angiogenesis (Figure 1). Multiple groups have built upon this work and shown how endometrial EVs from endometriotic lesions can abnormally enhance angiogenesis beyond the levels of endometrial EVs from healthy women.

Harp et al. demonstrated that EVs derived from endometriotic lesions can further enhance angiogenesis when added to primary human endothelial cells in vitro compared to adding EVs derived from a healthy endometrium. Their study showed that this enhanced angiogenesis correlated with an increase in expression of miR-21, a pro-angiogenic miRNA, within EVs from endometriotic lesions compared to the normal endometrium [41]. 

Hsu et al. also demonstrated that EVs derived from the ectopic endometrium enhanced angiogenesis more profoundly than EVs derived from the eutopic endometrium from the same patient. The researchers performed mass spectrometry to identify differential EV protein cargoes between the tissue specimens and found that annexin A2 was the most prominent difference, only being found in ectopic but not eutopic EVs. They suggested that annexin A2, as an EV cargo, plays a role in regulating endometriotic angiogenesis. They reported increased angiogenesis upon adding eutopic EVs when they were transfected with an Annexin A2 plasmid and a decrease in angiogenesis when they knocked down Annexin A2 within ectopic EVs [43].

Sun et al. built upon these observations and showed that angiogenic capabilities are even increased in EVs from the eutopic endometria of endometriosis patients compared to healthy patients’ endometria. They demonstrated an increase in neuroangiogenesis through in vitro tube formation and neurite outgrowth assays. They proved that this effect took place through EVs by blocking their secretion and showing that the pro-neuroangiogenesis effects decreased [72].

Khalaj et al. also showed increased pro-angiogenic and pro-inflammatory capabilities of EVs from endometriotic lesions compared to healthy patients. They went on to describe the unique miRNA-lncRNA signature within endometriotic EVs that distinguished them from their healthy counterparts. They postulated that this information could provide a basis for using EVs’ RNA profile as a biomarker of endometriosis [73].

An additional contributor to the spread of endometriotic lesions is the immune microenvironment of the uterus and peritoneal fluid [74]. In another report from Sun et al., they described that EVs may influence the immune microenvironment in the peritoneal fluid [75]. During endometriosis, macrophages located in the peritoneal cavity have been found to be preferentially polarized into the “alternately activated” anti-inflammatory M2 macrophage. This leads to an environment permissive of endometriotic lesion formation outside of the uterus [76]. Sun et al. collected EVs derived from lesions of a murine model for endometriosis. They treated macrophages in culture with these EVs and found that the macrophages were significantly polarized to the M2 phenotype, and their phagocytic ability was drastically decreased. They also found an increase in lesions when they treated their mice with these EVs [75].

During endometriosis, ectopic endometrial EVs contribute to an anti-inflammatory environment in the peritoneal fluid. However, at earlier stages of endometriosis progression, ectopic endometrial EVs have also been found to promote inflammation, which has been shown to drive the migration of endometrial tissue. Zhang et al. discovered that the expression of the lncRNA HOTAIR was increased in ectopic EVs. HOTAIR within EVs was shown to downregulate miR-761 expression, which ultimately led to activation of STAT3-related proinflammatory cytokines. They went on to show that this promoted angiogenesis and furthered the progression of endometriosis [77].

Collectively, these results indicated that aberrant EV signaling by both the eutopic and ectopic endometrium occurs in endometriosis, and this may contribute to the disease.

### 5.2. Adenomyosis

Adenomyosis is another condition that displays similar symptoms to endometriosis. However, instead of endometriotic lesions growing outside of the uterus, endometrial tissue is found abnormally embedded into the myometrium [78]. It is often characterized by symptoms such as uterine enlargement, infertility, and dysmenorrhea [79]. Epithelial-to-mesenchymal transition (EMT) of endometrial epithelial cells has been suggested to be a cause of adenomyosis [80]. EVs have been previously documented to be associated with EMT, especially as part of their involvement in cancer biology [81,82]. 

Chen et al. performed experiments to characterize EVs derived from adenomyotic lesions, and proteomic analysis revealed that a key protein involved with the EMT process, heat shock protein beta-1 (HSPB1), was contained within adenomyotic EVs. They further showed that endometrial epithelial cells were able to uptake adenomyotic EVs and that this induced an EMT-like event, which was identified by an increase in invasiveness and changes to cadherin expression [83].

Like endometriosis, the immune microenvironment plays a major role in the development of adenomyosis, with an accumulation of M2 macrophages being found in the endometria of adenomyotic patients [84]. Adenomyotic M2 macrophages have been shown to induce EMT in endometrial cells, furthering the progression of adenomyosis [85]. Hu et al. described that EVs isolated from the eutopic endometria of adenomyotic patients were able to induce polarization of macrophages into the M2b phenotype. Additionally, when these treated macrophages were co-cultured with endometrial epithelial cells, EMT was induced, and the migratory abilities of the epithelial cells increased [86].

EVs may also play a major role in some of the adverse reproductive outcomes that are characteristic of women with adenomyosis. Juárez-Barber et al. isolated EVs from the eutopic endometria of women with adenomyosis. They identified several miRNAs contained within the EVs that may promote implantation failure, including hsa-miR-24-3p and hsa-miR-423-5p, which have been associated with inhibiting trophoblast invasion. Additionally, they found hsa-miR-21-5p and hsa-miR-320a-3p, which are related to recurrent implantation failure [87]. 

### 5.3. Infertility and In Vitro Fertilization Failure

Infertility is a common condition affecting 12% to 18% of couples in the United States, and in vitro fertilization (IVF), or assisted reproductive technology (ART), is a common option to combat this condition [88]. During IVF, the embryo is fertilized outside the womb and is reinserted to begin implantation. Hormonal treatment to mimic a natural pregnancy is given to the patient prior to implantation, so the attempted implantation will begin during the “window of receptivity” of the endometrium. Earlier we discussed the important role that endometrial EVs play during embryo implantation. Endometrial EVs regulate endometrial receptivity and provide a mechanism of communication to the implanting embryo. This makes endometrial EV regulation an essential factor in the success of IVF.

Giacomini et al. analyzed the RNA cargo profile of EVs taken from the uterine fluid of fertile women compared to women undergoing ART [89]. They saw a significant correlation between the RNA cargo profile of UF-EVs and the transcriptomic profile of biopsies taken from the corresponding endometrium, suggesting the majority of UF-EVs originate from the endometrium. They also showed that there was a significant difference in the transcript levels of 2247 genes isolated from UF-EVs taken in the non-receptive phase compared to the receptive phase, again confirming a vital role for EVs from the endometrium. Finally, they showed a significant change in the transcript levels of 161 genes in UF-EVs of IVF patients with successful versus failed implantation. They also showed that UF-EVs isolated from patients with successful implantation were slightly, but significantly smaller in size [89]. Together these data suggest the importance of UF-EVs in regulating embryo implantation, especially in IVF patients.

Li et al. performed a similar analysis, but they focused on small non-coding RNA (sncRNA) content of UF-EVs from healthy women and women undergoing IVF during the receptive and non-receptive stages of the endometrium. They found 12 sncRNA that were strongly associated with biological functions crucial for implantation, such as extracellular matrix, immune response, and cell junction, and these sncRNA were conserved in both healthy and IVF patients. They also showed that IVF patients who did not conceive showed differential sncRNA expression within their EVs compared to patients who had successful implantation and pregnancy. One miRNA in particular, hsa-miR-262-3p, was robustly overexpressed in patients that were unable to conceive [90]. 

Although they did not specifically study IVF patients, Rai et al. also showed via proteomics that the protein cargoes of EVs isolated from uterine lavage change depending on the receptive or non-receptive phase of the endometrium, with an enrichment of invasion-relevant proteins present in EVs from the receptive phase. They also showed that EVs from fertile women contain proteins significant for embryonic implantation, such as ANXA2, PRDX2, SERPING1, and IDHC, that are absent in samples from infertile women [44].

These studies suggest a strong correlation between IVF implantation success and the protein or sncRNA content of UF-EVs of the patient. IVF is an expensive procedure, with each cycle costing approximately USD 19,200 and multiple cycles often needed for a successful pregnancy [91]. A successful pregnancy is still not guaranteed, and the cost barrier prevents many couples from accessing this option. Since its development in 2011, an endometrial receptivity array (ERA) performed on an endometrial biopsy is sometimes used prior to embryo transfer to identify the window of endometrial receptivity based on the transcriptomic profile of implantation-significant genes in the endometrium [92,93,94]. These studies suggest a potentially more relevant and less invasive test to predict implantation success. A “liquid biopsy” of UF-EVs could provide vital information to the patient on the chances of a successful implantation, allowing for an informed decision from the couple without an invasive biopsy, and could prevent a potential financial and emotional burden on the couple if the EV profile suggests low chances of implantation success.

### 5.4. Endometrial Cancer

Endometrial cancer is responsible for more than 89,000 deaths each year and is one of the most common cancers found in women [95]. As with other types of cancer, EV signaling has been shown to play a role during endometrial cancer. Roman-Canal et al. isolated EVs from a peritoneal lavage of healthy or endometrial cancer patients. They profiled the miRNA content of the EVs and identified 114 miRNAs that were significantly dysregulated between the disease and control conditions. Several of these miRNAs are associated with tumor progression in both endometrial cancer and other cancer types. They posited that this EV miRNA profile could be a valuable source of biomarkers for early detection of cancer [96]. In a similar study, Xu et al. isolated EVs from serum samples of endometrial cancer patients and profiled the circular RNA (circRNA) within the EVs. They showed significant changes in 275 circRNAs when compared to healthy controls [97]. In fact, EVs associated with endometrial cancer have been called a “biomarkers treasure trove” due to their distinct changes in miRNA when compared to healthy patients [98].

### 5.5. Environmental Toxicants and Endometrial EV Signaling

Environmental exposure to toxicants, such as air pollution, cigarette smoke, metals, and manufacturing chemicals, contributes to the pathogenesis of various diseases, including those within the reproductive tract. There is increasing evidence that changes in EV signaling upon environmental toxicant exposure is likely to be associated with pathophysiological conditions. These could be due to alterations in the quantity of EVs secreted from various tissues and changes in the protein and nucleic acid cargo of the EVs [99]. 

Shepherd et al. investigated how EVs derived from decidualized endometrial stromal cells had altered protein cargoes in response to treatments of cigarette smoke. They showed that cigarette-smoke-treated decidual cells produced EVs with more inflammatory-related proteins than the control. They went on to prove that the maternal EVs could be taken up by trophoblast cells and that cigarette-smoke-treated EVs caused an increased inflammatory response by the trophoblast cells [100]. Feto-maternal inflammation can be a key trigger mechanism for preterm birth [101]. 

Exposure to metals is also known to increase risk for pregnancy complications such as preterm birth, fetal growth restriction, and gestational diabetes [102,103,104]. Howe et al. performed an epidemiological study where they showed an association between urinary metal levels and maternal EV miRNA in pregnant women. They found eight miRNAs within maternal EVs that were positively associated with three different metals (Ba, Hg, and Tl). Seven of these eight miRNAs are associated with the EGFR pathway, which plays an important role in endometrial function during early pregnancy, and five of the eight are also enriched in the VEGF pathway, which also plays a role during placental angiogenesis [105].

## 6. Conclusions and Future Directions

Throughout pregnancy, endometrial extracellular vesicles play essential roles in guiding embryo implantation, facilitating maternal–fetal communication, and promoting the development of uterine vasculature to ensure successful gestation. EVs function as a vehicle for shuttling key bioactive cargo such as protein and miRNA between various cell types within the pregnant uterus, creating a harmonious microenvironment conducive to embryonic growth and development. 

The processes induced by EVs seem to be largely dependent upon their cargo. Heterogeneity among EV cargoes secreted by the endometrium allows for EVs to coordinate multiple significant functions during pregnancy. The regulatory pathways which direct endometrial EVs to specific tissues, leading to distinct effects at appropriate timepoints, are still not completely understood. However, it is evident that hormonal regulation by estrogen and progesterone promotes endometrial EV secretion and alters the EV cargo. Further, a clear understanding of how hormones and other factors regulate endometrial EV secretion and cargo composition to initiate different processes in a time- and tissue-sensitive manner remains necessary, and this should be a major area of research moving forward.

Disruptions in the delicate balance of endometrial EV secretion and cargo content can have profound pathological consequences. Dysregulation of these vesicles has been associated with a variety of gynecological conditions, such as endometriosis, infertility, endometrial cancer, and preeclampsia. As we continue to find links between aberrant EV signaling and pregnancy and uterine-related pathologies, it becomes evident that endometrial EVs hold significant promise as diagnostic biomarkers and therapeutic targets for these conditions. Further analysis of the underlying molecular mechanisms governing their biogenesis, cargo selection, and tissue uptake could pave the way for the development of innovative interventions to prevent or treat these disorders.

## Figures and Tables

**Table 1:** Partial list of significant proteins and miRNAs identified as cargoes of endometrial EVs.

EV Cargo	Cargo Type	Potential Event Influenced	Species	Reference
hsa-miR--100	miRNA	Endometrial receptivity, blastocyst implantation	Human	[38]
hsa-miR-193a-5p	miRNA	Endometrial receptivity, blastocyst implantation	Human	[38]
hsa-miR-30b	miRNA	Endometrial receptivity, blastocyst implantation	Human	[38]
hsa-miR-30d	miRNA	Endometrial receptivity, blastocyst implantation	Human, Sheep	[38,39,40]
hsa-miR-31	miRNA	Endometrial receptivity, blastocyst implantation	Human, Sheep	[38,40]
hsa-miR-452	miRNA	Endometrial receptivity, blastocyst implantation	Human	[38]
hsa-miR-455-3p	miRNA	Endometrial receptivity, blastocyst implantation	Human	[38]
miR-126	miRNA	Angiogenesis	Human	[41]
miR-21	miRNA	Angiogenesis	Human	[41]
ssc-let-7a	miRNA	Endometrial receptivity, blastocyst development	Pig, Sheep	[40,42]
ssc-let-7g	miRNA	Endometrial receptivity	Pig	[42]
Angiopoietin-1	Protein	Angiogenesis	Human	[30]
Angiopoietin-related protein 2	Protein	Angiogenesis	Human	[30]
Annexin A2	Protein	Angiogenesis, endometrial receptivity	Human	[30,31,43,44]
Decorin	Protein	Decidualization, angiogenesis	Human, Mouse	[30,35,44]
ERAP-1 (endoplasmic reticulum aminopeptidase 1)	Protein	Angiogenesis	Human	[44,45]
Gap Junction A1	Protein	Angiogenesis	Human	[30,44]
Glucose Transporter 1	Protein	Decidualization	Human, Mouse	[30,35,44]
Insulin-like Growth Factor 2	Protein	Trophoblast differentiation, angiogenesis	Human	[30]
Insulin-like Growth Factor Binding Protein 1	Protein	Trophoblast differentiation	Human	[30,44]
Insulin-like Growth Factor Binding Protein 7	Protein	Trophoblast differentiation	Human	[30,44]
Isocitrate Dehydrogenase 1	Protein	Blastocyst implantation	Human, Sheep	[40,44]
Lactadherin	Protein	Decidualization, angiogenesis	Human, Mouse	[30,31,35,44]
MMP2 (72 kDa type IV collagenase)	Protein	Angiogenesis	Human	[30,44]
Peroxiredoxin 2	Protein	Blastocyst implantation	Human	[30,44]
Pyruvate Kinase Mutase	Protein	Decidualization	Human, Mouse	[30,35,44]
Serpin Family G Member 1	Protein	Endometrial receptivity	Human	[30,44]

## Data Availability

No new data were created or analyzed in this study. Data sharing is not applicable to this article.
